# ^11^C‐PK11195 PET imaging and white matter changes in Parkinson’s disease dementia

**DOI:** 10.1002/acn3.50877

**Published:** 2019-09-10

**Authors:** Nicolas Nicastro, Ajenthan Surendranathan, Elijah Mak, James B. Rowe, John T. O’Brien

**Affiliations:** ^1^ Department of Psychiatry University of Cambridge Cambridge United Kingdom; ^2^ Department of Clinical Neurosciences Geneva University Hospitals Geneva Switzerland; ^3^ Department of Clinical Neurosciences University of Cambridge Cambridge United Kingdom

## Abstract

There is evidence of increased microglial activation in Parkinson’s disease (PD) as shown by in vivo PET ligand such as ^11^C‐PK11195. In addition, diffusion tensor imaging (DTI) imaging reveals widespread changes in PD, especially when the associated dementia develops. In the present case series, we studied five subjects with Parkinson’s disease dementia (PDD). Our findings suggest that while DTI metrics mirror cognitive severity, higher ^11^C‐PK11195 binding seems to be associated with a relative preservation of both white matter tracts and cognition. Longitudinal studies are warranted to tackle the complex relationship between microglial activation and structural abnormalities in neurodegenerative conditions.

## Introduction

Neuroinflammation is increasingly recognized as a key contributor to neurodegeneration in Parkinson’s disease (PD) and dementia.^1^ PET probes have been developed to assess in vivo brain inflammation, for example, by targeting translocator protein (TSPO).^2^ Among those, ^11^C‐PK11195 is one of the most widely used ligands to estimate microglial activation.^3^ Recent studies in Alzheimer’s disease (AD) and dementia with Lewy bodies (DLB) have shown that microglial activation, as measured with ^11^C‐PK11195 PET, is more prominent in patients at an earlier disease stage.^4^, ^5^ Diffusion tensor imaging (DTI), on the other hand, is an application of diffusion‐weighted magnetic resonance imaging designed to assess microstructural integrity based on motion of water molecules. Impaired white matter integrity in people with PD has been consistently observed in corpus callosum, in relation to cognitive impairment and development of dementia.^6^, ^7^ In the present case series, we studied ^11^C‐PK11195 binding as an index of neuroinflammation, and its association with DTI, motor, and cognitive features of Parkinson’s disease dementia (PDD).

## Subjects and Methods

We studied five PDD subjects who were recruited within the Neuroimaging of Inflammation in Memory and other Disorders (NIMROD) study protocol.^8^ Demographics are available in Table 1. Each subject underwent structural MRI, 3T DTI, and ^11^C‐PK11195 PET imaging, as well as in‐depth neuropsychological and motor assessment, including Addenbrooke's Cognitive Evaluation Revised (ACER) and Movement Disorders Society (MDS) Unified Parkinson’s Disease Rating Scale (UPDRS) part III motor assessment.

**Table 1:** Demographics, clinical, and imaging features of included PDD subjects.

Subject	Age (years), Gender	MDS‐UPDRS III, *ON‐state*	ACER	DTI global radial diffusivity	Significant ^11^C‐PK11195 BP_ND_ changes compared to Controls
PDD 1	78, M	36	86	5.11 × 10^−4^	Putamen (↑), substantia nigra (↓)
PDD 2	70, M	40	80	5.36 × 10^−4^	Parahippocampal gyrus (↓)
PDD 3	70, M	48	76	6.19 × 10^−4^	Substantia nigra (↓)
PDD 4	81, M	36	69	6.09 × 10^−4^	Midbrain (↓), medulla (↓)
PDD 5	68, M	34	85	4.83 × 10^−4^	Mesial anterior temporal lobe (↓), fusiform gyrus (↓), superior parietal gyrus (↓), lateral orbital gyrus (↓), amygdala (↓), nucleus accumbens (↓), substantia nigra (↓), pons (↓)
Mean ± SD	73.4 ± 5.7	38.8 ± 5.6	79.2 ± 7.0	5.52 × 10^−4^	

Mean cortical thickness was obtained with Freesurfer 6.0 using the standard processing pipeline described in.^9^ DTI imaging was processed with FSL 6.0.^10^ Briefly, this included registration of all diffusion‐weighted images to the *b* = 0 (i.e., no diffusion) volume using the FMRIB Software Library (FSL) Diffusion Toolbox, followed by brain masks creation with *Brain Extraction Tool*, head movement, and eddy currents correction. We then used *DTIfit* to independently fit the diffusion tensor for each voxel, resulting in the derivation of fractional anisotropy (FA), mean and radial diffusivity (MD and RD, respectively).

Details about ^11^C‐PK11195 PET processing are available in^4^. In brief, binding in each region of interest was quantified using non‐displaceable binding potential (BP_ND_) determined with a simplified reference tissue model incorporating vascular binding correction and reference region time‐activity curve estimation from supervised cluster analysis using four kinetic classes. Regional BP_ND_ was corrected for CSF contamination through division of the region of interest time‐activity curve with the mean region of interest fraction of grey and white matter. Regional BP_ND_ binding was obtained for each subject with the Hammers atlas.^4^, ^11^

Subsequently, regional ^11^C‐PK11195 binding Z‐scores were computed by comparing each PDD subject’s values to 16 similarly aged control subjects (mean ± SD age 69.7 ± 6.6 years, 50% female participants, ACER score 92.5 ± 5.6) scanned with the same imaging protocol,^2^ with *Z*‐scores higher/lower than 2.0 considered as significant. Spearman correlations and regression analyses were performed in the PDD group between composite lobar (frontal, temporal, parietal, occipital) and whole‐cortex ^11^C‐PK11195 BP_ND_, DTI metrics, and clinical data. Given the exploratory design of the study, results were not corrected for multiple comparisons.

## Results

**Figure 1:**
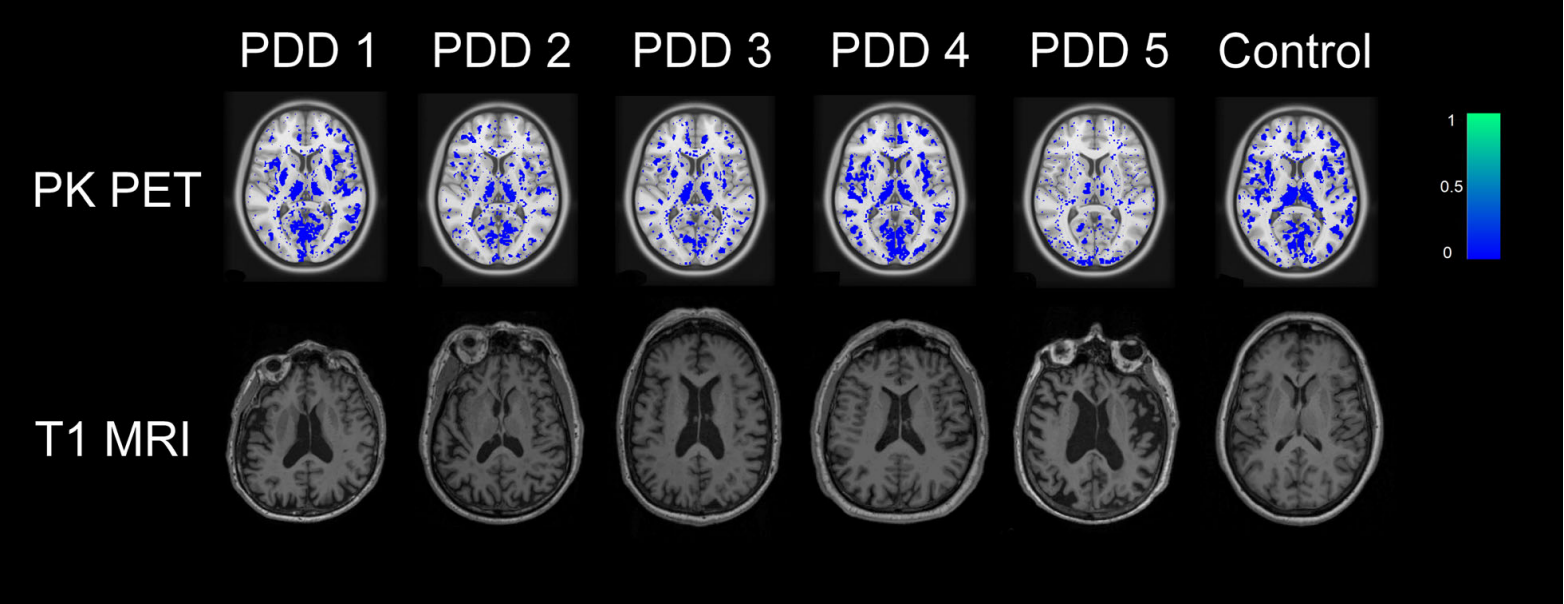
Axial ^11^C‐PK11195 BP_ND_ images and T1‐weighted structural MRI of the five PDD included in the study and one representative control subject (65‐year‐old female participant, ACER 96/100). PDD patients have similar ^11^C‐PK11195 binding than the control participants, except PDD 5 who has lower binding values in several regions (cf. Table 1).

Demographics and main findings are available in Table 1. The analyses revealed that PDD subjects had comparable or lower levels of whole‐cortex as well as regional ^11^C‐PK11195 binding relative to controls (Fig. 1). In addition, lobar occipital ^11^C‐PK11195 binding was inversely correlated with MD and RD after adjusting for mean cortical thickness (*linear regression*, for MD: *B* = −415.6, *P* = 0.035, adjusted *R* ^2^ = 0.89; for RD: B coefficient = −348.6, *P* = 0.032, adjusted *R* ^2^ = 0.90), that is, higher ^11^C‐PK11195 was associated with a relative preservation of white matter integrity. The directionality of these PET‐MRI correlations was congruent with those from neuropsychological data. In fact, ACER score positively correlated with frontal ^11^C‐PK11195 binding when adjusting for mean cortical thickness (*linear regression*, *B* = 641.2, *P* = 0.014, adjusted *R* ^2^ = 0.95). In addition, there was a trend for higher ACER scores being related to preserved DTI metrics (lower MD/RD and higher FA, Spearman *P* = 0.10, rho = −0.80 for MD/RD and 0.80 for FA). There was no significant association between MDS‐UPDRS part III score and ^11^C‐PK11195 binding or DTI metrics.

## Discussion

Our study suggests that higher ^11^C‐PK11195 binding is associated with a relative preservation of both white matter tracts and cognition. Specifically, we found that DTI metrics mirror disease severity, and that PDD subjects had similar or reduced ^11^C‐PK11195 binding compared to controls.^6^, ^12^ While these findings require confirmation with larger samples, longitudinal data, and finer imaging proxies (e.g., voxel‐wise DTI association with ^11^C‐PK11195 binding), this case series brings novel evidence for an early role of neuroinflammation in the pathophysiology of PDD and confers additional support for the hypothesis that central inflammation represents a potential therapeutic target in dementia.

## Author Contributions

Nicolas Nicastro contributed to research project execution, data review and critique, and manuscript draft. Ajenthan Surendranathan also contributed to research project execution, data review, and manuscript review. Elijah Mak contributed to research project execution, data review, and manuscript review. James B. Rowe contributed to research project conception, organization and execution, data review, and manuscript review. John T. O’Brien contributed to research project conception, organization and execution, data review, and manuscript review.

## Conflict of Interest

The authors have no conflict of interest to report.
